# Buffalo Medical and Surgical Association

**Published:** 1890-03

**Authors:** W. H. Bergtold

**Affiliations:** Secretary


### BUFFALO MEDICAL AND SURGICAL ASSOCIATION.

Reported by W. H. BERGTOLD, M. D., Secretary.

Regular monthly meeting held in Room No. 1, Y. M. C. A. Building, West Mohawk street, January 7th, at 8:30 P. M.

The president, Dr. A. A. HUBBELL, in the chair.

Drs. FLAGG and CONGDON were elected to membership.

Dr. CHARLES G. STOCKTON then read on Some Phases of Glycosuria.

DISCUSSION.

Dr. J. W. GROSVENOR.—In regard to the treatment of diabetes, personally he directs his treatment mainly to the nutrition of his patient, and employs the so-called diabetic bread. Those patients who pay the most attention to their digestion seem to do the best.

Dr. HOPKINS was glad to have the essayist present this view of the cause and treatment of glycosuria, for it, in a manner, substantiated what he had testified to in an important "Will Case," not many years ago. In the case in question, the patient had daily consumed large quantities of champagne, carbonated waters, etc. Some of the most eminent foreign physicians prognosticated that he would not live longer than fifteen months; whereas, the man survived that many years.

Dr. COAKLEY spoke approvingly of the hygienic and dietary measures usually adopted to-day. He also thought that exercise was very essential. He asked whether Dr. Stockton could state the amount of the diminution of carbonic oxide in the tissues.

Dr. STOCKTON, in closing, remarked that, in his belief, the so-called "diabetic breads" were useless, and a snare. He did not recall the exact diminution of $CO^2$ in the tissues.

Dr. H. R. HOPKINS then read a paper, entitled, Treatment of Some of the Local Manifestations of Phthisis. He called attention to the fact that phthisis pulmonalis was curable, as evinced by the scars of healed cavities. Pepper first drew attention to the fact, that a needle, under proper conditions, could be thrust into the living lung with no ill effects. Dr. Hopkins wished to call attention to the method of treating pulmonary cavities by injection. He used creasote, dissolved in almond oil, and employed a subcutaneous injection of cocaine to lessen the pain of the needle-puncture. The results of his personal work were then related. In one case death resulted from the injection needle penetrating an emphysematous area, and on the withdrawal of the needle pneumothorax followed, causing dyspnea through extensive disease of the other lung, which soon resulted in death. Dr. Hopkins hoped to continue his experiments, and would give a later report when possible.

DISCUSSION.

Dr. TREMAINE failed to see that any of the cases had been benefited. It is a difficult task to locate cavities, and this alone weighs against the treatment. Pure mountain air gives much better results.

Dr. BERGTOLD, having made the autopsy on the case mentioned by Dr. Hopkins, drew attention to certain conditions not mentioned by the essayist. The injection was given by a hospital interne who selected the wrong lung, which, aside from patches of emphysema, was fairly healthy. The diseased lung was practically of no use, and when the pneumothorax supervened the patient fell an easy prey to it.

Dr. STOCKTON mentioned a case (of a woman) where a pneumothorax ensued after inter-pulmonary injection, and where the patient also died. This liability constitutes quite an objection, in his estimation, against this method of treatment. Personally, he does not favor it, but anything which offers even a chance only for the cure of phthisis, ought to be thoroughly and well investigated.

Dr. KRAUSS believed that this method of treatment is of little use, because of the nasty condition of the cavity wall, and was of the opinion that if we could clean the cavities out, we would get better results.

Dr. HOPKINS, in closing, remarked that some one has said that for phthisis, nothing can be given but "Lies and Morphine." We must be patient, and not expect too great results at once. He recalled two cases, particularly, where every one gave an absolutely hopeless prognosis, yet, on the institution of this treatment, a steady improvement, and ultimate recovery were had. How much was due to the injections, he does not claim to estimate.

Dr. M. D. MANN stated that he had made three laparatomies during the day of the meeting, and that two were quite interesting, presenting specimens from these cases. The first was that of a young woman who has suffered very much from dysmenorrhea, and in whose case he diagnosticated an enlarged ovary behind the uterus as the cause of the trouble. On operating, he was able to remove an ovary as diagnosed. It was converted into a dermoid cyst. The second was that of a woman thirty years old, who had been infected with gonorrhea by her husband, during the latter months of pregnancy. On the second day after confinement she had a chill, and other evidances of pelvic inflammation. This continued for four months, when the woman was practically a wreck, had pain all the time, and could be out of bed but a short time each day. Examination showed a great enlargement and tenseness of the pelvic tissues behind, and at the sides of the uterus. Diagnosed pyosalpinx. On operating, he found each tube closed and filled with pus; the ovaries also contained collections of pus. Gonorrheal puerperal fever, in this case, came on very early, and it is all the more interesting, because of so few cases having been reported when the disease appeared so early.

Essayists for the February 4th meeting announced as Dr. W. S. Renner, subject, "Adenoid Vegetations of the Naso-Pharynx, and Dr. M. D. Mann, "One hundred and fifty Cases of Abdominal Section with Reference to Prevention of Complications."

